# Notch-Wnt signal crosstalk regulates proliferation and differentiation of osteoprogenitor cells during intramembranous bone healing

**DOI:** 10.1038/s41536-021-00139-x

**Published:** 2021-05-28

**Authors:** S. Lee, L. H. Remark, A. M. Josephson, K. Leclerc, E. Muiños Lopez, D. J. Kirby, Devan Mehta, H. P. Litwa, M. Z. Wong, S. Y. Shin, P. Leucht

**Affiliations:** 1 Department of Orthopaedic Surgery, NYU Robert I. Grossman School of Medicine, New York, NY USA; 2 Institute of Comparative Molecular Endocrinology, Ulm University, Ulm, Germany; 3 Department of Cell Biology, NYU Robert I. Grossman School of Medicine, New York, NY USA

**Keywords:** Regeneration, Mesenchymal stem cells

## Abstract

Adult bone regeneration is orchestrated by the precise actions of osteoprogenitor cells (OPCs). However, the mechanisms by which OPC proliferation and differentiation are linked and thereby regulated are yet to be defined. Here, we present evidence that during intramembranous bone formation OPC proliferation is controlled by Notch signaling, while differentiation is initiated by activation of canonical Wnt signaling. The temporospatial separation of Notch and Wnt signal activation during the early stages of bone regeneration suggests crosstalk between the two pathways. In vitro and in vivo manipulation of the two essential pathways demonstrate that Wnt activation leads to initiation of osteogenic differentiation and at the same time inhibits Notch signaling, which results in termination of the proliferative phase. Here, we establish canonical Wnt signaling as a key regulator that facilitates the crosstalk between OPC proliferation and differentiation during intramembranous, primary bone healing.

## Introduction

Injuries to the musculoskeletal system are the most common cause for disability. While the majority of fractures heal without sequelae, about 5% of fractures result in non-union, which subjects patients to pain, prolonged immobility, and revision surgery^1^. To this point, there is no biological adjuvant for fracture non-unions that reliably promotes fracture healing, alone or in combination with revision surgery.

The burgeoning field of stem cell biology is an unambiguous indicator of society’s interest in the regenerative potential of the human body and new evidence reveals that stem cells derived from adult tissues have the capacity to differentiate into a multitude of cell types^2^. This regenerative potential of adult stem cells suggest that these cells may be used in the future to fulfill the mounting needs of patients with degenerative disease and traumatic tissue loss. There are, however, significant gaps in our understanding of stem cell biology that impede their widespread use in treating skeletal conditions. For example, signaling pathways that control proliferation and differentiation have been identified, but we are still in the early stages of understanding the fine-tuned interplay between these individual pathways. During skeletal repair this switch from proliferation to differentiation is especially critical since the total number of available skeletal stem and progenitor cells is fairly small (0.3% of all bone marrow cells^3,4^ in relation to the often-encountered size of the bony defect. Thus, proliferation represents the mechanism by which the injury side is provided with a sufficient number of osteogenic or chondrogenic cells that will ultimately result in bone formation and restoration of limb function.

Notch signaling is an evolutionarily conserved signaling pathway in the development of multicellular organisms. Its temporal-spatial expression specifies diverse cellular events including proliferation, differentiation, apoptosis, stem cell self-renewal, and binary cell-fate specification^5,6^. There are four Notch receptors (Notch 1–4) and multiple ligands (Jagged1–2, DLL1,3,4). Notch signaling requires cell-cell contact between the ligand and the receptor, which in turn results in activation of the Notch intracellular domain (NCID), a transcription complex that traffics into the nucleus. Downstream targets include Hes-1and 5 (Hairy Enhancer of Split), Hey 1 and 2 (Hairy/enhancer-of-split related with YRPW motif protein)^7,8^. Recent studies have identified an important role of Notch signaling during skeletal development and bone homeostasis (reviewed in refs. ^9–12^), however little is known about the role of Notch signaling in skeletal regeneration and its potential integration and interaction with other pathways, such as the Wnt signaling pathway during this process.

Wnts act as long-range, concentration-dependent morphogens that bind to cell surface receptors encoded by the Frizzled and low-density lipoprotein receptor related proteins (Lrp). Once bound, Wnt ligands initiate a cascade of intracellular events that eventually lead to the transcription of target genes through the nuclear activity of beta-catenin and the DNA binding protein TCF (reviewed in refs. ^13–15^). Wnts are involved in a wide variety of cellular decisions associated with the program of osteogenesis^16–18^. For example, Wnts regulate the expression level of *sox9*^19^, which influences the commitment of mesenchymal progenitor cells to a skeletogenic fate^20,21^. In adult animals there is abundant evidence that Wnt signaling regulates bone mass (reviewed in ref. ^22^). For example, mutations in the human Wnt co-receptor LRP5 are associated with several high bone mass syndromes including osteopetrosis type I, and endosteal hyperostosis or autosomal dominant osteosclerosis, as well as a low bone mass disease, osteoporosis-pseudoglioma^23–25^.

Youngstrom et al. postulate that there is a temporospatial fate switch that occurs during the continuum of bone regeneration^26^, however, what regulates this molecular switch is still unknown. We aimed to fill this knowledge gap by investigating how the two pathways, Notch and Wnt signaling, cooperatively orchestrate early osteoprogenitor cell proliferation and differentiation during intramembranous bone healing. While much effort has been exerted into identifying the effect of a singular growth factor on bone regeneration, our research seeks to characterize the well-coordinated interplay between two pathways to further develop biologic therapeutics that mimic the physiology of bone regeneration.

Here, we dissect the temporal and spatial role of Notch and Wnt signaling during intramembranous bone formation, and by using molecular pathway manipulation, we identify an interaction between the two pathways resulting in a programmatic switch from proliferation to differentiation.

## Results

### Temporal separation of proliferation and differentiation during bone regeneration

**Fig. 1:**
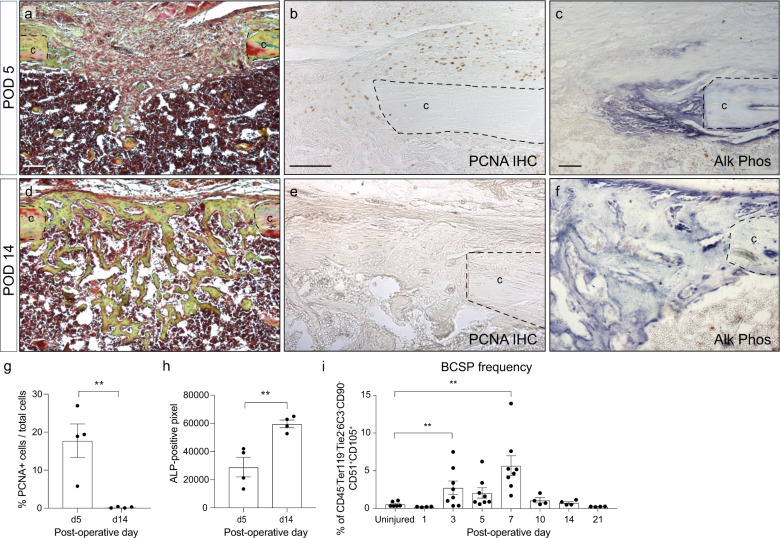
Temporal separation of proliferation and differentiation during tibial defect healing. **a** Histological section of tibial mono-cortical defects 5 days after injury, stained with Movat’s Pentachrome. The defect site is filled with soft tissue. **b** PCNA IHC shows active proliferation in the periosteum and defect site at this early time point. **c** Alkaline phosphatase staining demonstrates only a small region of osteogenic differentiation at the cortical edge. **d** After 14 days, the defects site is filled with woven bone, stained yellow-green with Pentachrome. **e** PCNA staining reveals absence of proliferation within the injury. **f** Alkaline phosphatase staining indicating osteogenic differentiation throughout the injury site. **g** Quantification of PCNA-positive cells in the injury site at POD 5 and 14. **h** Quantification of alkaline phosphatase staining at POD 5 and 14. **i** Frequency of bone-cartilage-stromal progenitor cells (BCSPs) was analyzed over the time course of fracture healing by flow cytometry using the following BCSP markers: CD45^−^ Ter119^−^ Tie2^−^ CD51^+^ CD90^−^ 6C3^−^ CD105^+^. Scale bar = 100 μm. ALP alkaline phosphatase, BCSP bone-cartilage-stromal progenitor cells, c cortical bone, PCNA proliferating cell nuclear antigen, POD postoperative day. **p < 0.01. Data were represented as mean ± s.e.m.

On a cellular level intramembranous bone healing is characterized by an early proliferate phase (Fig. 1a–c, g), which is followed by a wave of differentiation that leads to bony callus formation and ultimately healing of the fracture with restoration of mechanical integrity (Fig. 1d–f, h). During the early proliferative phase (through postoperative day 7), bone-cartilage-stromal-progenitor cell (BCSP)^27–29^ number increases until differentiation ensues, at which point the number of BCSPs within the regenerate returns to baseline (Fig. 1i and Supplementary Fig. 1). Here, we set out to uncover the molecular switch that regulates proliferation and differentiation during intramembranous bone healing.

### Notch signaling is active during intramembranous bone healing, followed by activated Wnt signaling

**Fig. 2:**
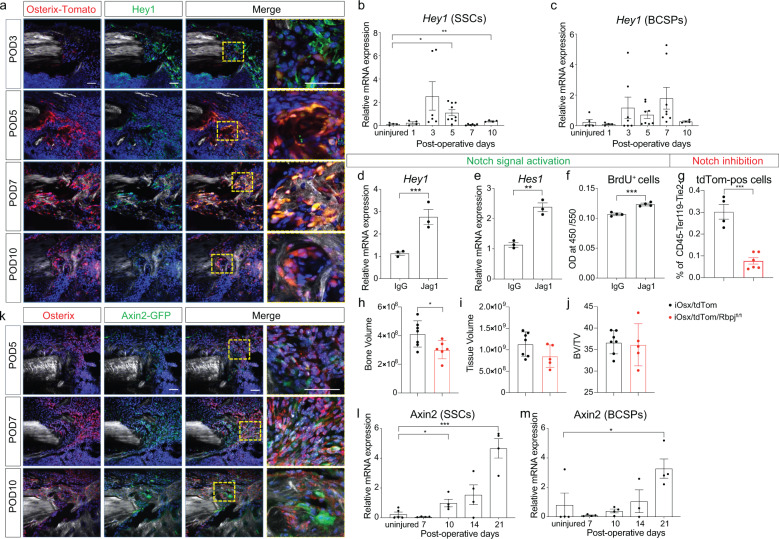
Notch signaling dominates the proliferative phase while canonical Wnt signaling governs the differentiation phase. **a** Temporospatial expression profile of Notch downstream target Hey1 by immunostaning and co-localization with osterix-positive cells. To label osterix-positive cells, iOsx/tdTom mice were pulsed by tamoxifen injection (1 mg/day/mouse) twice at 1 day before surgery and 1 day after surgery. **b**, **c** Temporal Notch downstream target gene expression during the early injury response (uninjured, POD 1–10) in SSCs and BCSPs (*n* = 6–9). **d**, **e** Notch activation using Jagged1-coated tissue culture plates resulted in upregulation of the Notch target genes *Hey1* and *Hes1*. **f** In response to Notch activation, proliferation increased, shown by BrdU quantification. **g** The number of tdTomato-positive (*osx*-positive OPCs) cells decreased in the POD 3 injury site after Notch inhibition in iOSX/tdTom/Rbpj^fl/fl^ mice. Tamoxifen (1 mg/day/mouse) was injected from day −1 until day 2. **h** Bone volume, **i** tissue volume, and **j** bone volume/total volume, at postoperative day 10 using microCT histomorphometry demonstrates a smaller bone volume in the regenerate in the iOSX/tdTom/Rbpj^fl/fl^ mice (tamoxifen (1 mg/day/mouse daily) from day −1 to day 6). **k** Spatial expression of Wnt responsiveness within *osx*-positive (IF) OPCs using iAxin2/GFP reporter mice (Tamoxifen administration: 1 day before euthanasia). Scale bar = 50 μm. **l**, **m** *Axin2* gene expression SSCs and BCSPs in uninjured and POD 7–21 days (*n* = 4). BCSP bone-cartilage-stromal progenitor cells, Jag1 Jagged1, POD postoperative day, SSC skeletal stem cell. *p < 0.05, **p < 0.01, ***p < 0.001. Data were represented as mean ± s.e.m.

There is ample evidence in the literature that Notch signaling regulates proliferation during tissue regeneration in a variety of tissues^30–34^, including bone^12,35^. It is yet unknown whether Notch signaling performs a similar function during intramembranous bone healing of the appendicular skeleton. Therefore, we screened the regenerate within the tibial defect site of tdTomato;Osx-creERT2 mice (iOsx/tdTom) at varying time points post-injury for the Notch downstream target Hey1 using immunofluorescence staining, and found that Hey1 was upregulated during the early, proliferative phase (Fig. 2a and Supplementary Fig. 2). While most of the Hey1-positive cells co-labeled with the osterix-positive and therefore the osteogenic lineage, we confirmed this finding in a more stem/progenitor cell specific assay. Skeletal stem cells (SSCs) and BCSPs were isolated from injury sites at 1, 3, 5, 7, and 10 days post injury, and *Hey1* expression levels were determined using qRT-PCR. Again, we observed increased expression of *Hey1* between day 3 and 10 in both skeletal stem and progenitor cells (Fig. 2b, c). RNAseq, FACS and immunofluorescence staining identified the Notch2 receptor as the most dominant receptor, while Jagged-1 was the most highly expressed ligand (Supplementary Fig. 3). These data provide convincing evidence that Notch signaling is active in skeletal stem and progenitor cells during the proliferative phase of intramembranous bone healing. Whether Notch activation causes proliferation of osteoprogenitor cells (OPCs) is yet unknown. We therefore activated Notch signaling in bone marrow stromal cells, grown on Jagged1-coated plates, which resulted in increased *Hey1* and *Hes1* expression, confirming Notch activation (Fig. 2d, e). This Notch activation led to an increased proliferation shown by BrdU (Fig. 2f), confirming the proliferative effect of Notch signaling. We then utilized a transgenic approach, in which Notch signaling is inhibited by conditional deletion of RBPJ^36^ in *Osx*-expressing cells. RBPJ is a major transcriptional effector of Notch signaling^37^ and when knocked out in *Osx*-expressing OPCs^38^, we observed a reduction in tdTom-positive cells, and thus *Osx*-expressing OPCs within the injury site at postoperative day 3, further confirming the effect of Notch signaling on proliferation (Fig. 2g). This reduction in the number of OPC manifested itself as a smaller bone volume (BV) in the regenerate at postoperative day 10 (Fig. 2h–j).

Wnt signaling has been shown to induce osteogenic differentiation during bone regeneration^39–42^, however, it has yet to be determined how the switch from proliferation to differentiation is orchestrated. Using *Axin2-cre*^*ERT2*^*/Rosa26-loxP-stop-loxP-eGFP* (iAxin2/GFP) reporter mice^43^, we surveyed the regenerate tissue at varying time points to establish a temporal profile of Wnt responsiveness. At postoperative day 5, only a few cells close to the cortical edge were labeled with GFP, while at day 7 and 10, the majority of *Osx*-positive cells within the defect site were GFP-positive, thus Wnt-responsive (Fig. 2k and Supplementary Fig. 2). Again, we isolated SSCs and BCSPs from the injury site by flow cytometry and confirmed the increase in Wnt signaling using *Axin2* expression as a proxy. Both SSCs and BCSPs exhibited increased *Axin2* expression during the differentiation phase starting at POD 10 (Fig. 2l, m).

### Wnt inhibition maintains Notch activation and prolongs the proliferative response

**Fig. 3:**
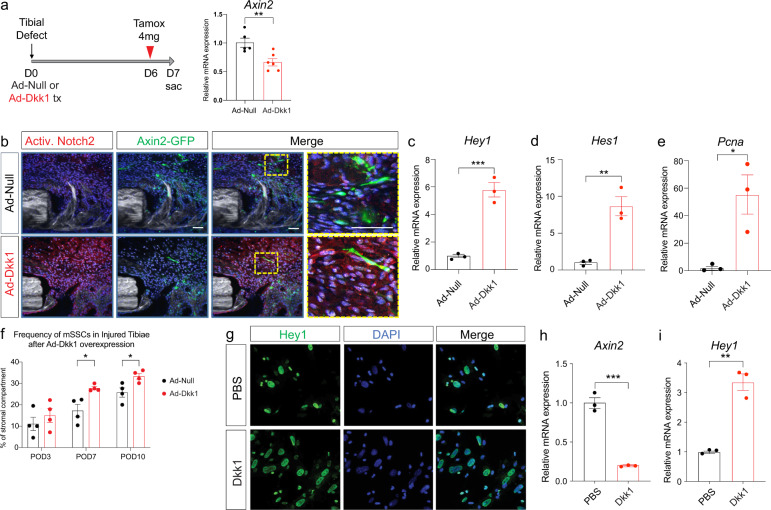
Wnt inhibition sustains Notch activation and lengthens the proliferative phase. **a** Ad-Null (control) or Ad-Dkk1 (Wnt antagonist) was administered around tibial defect sites of iAxin2/GFP reporter mice and tamoxifen was injected at POD 6 (24 h before euthanasia) (*n* = 5–6). *Axin2* expression levels were significantly decreased at day 3 after Ad-*Dkk1* administration, confirming Wnt inhibition. **b** Immunofluorescence staining against NICD2 (activated Notch2 intracellular domain) reveals near absent Notch activation in the control injury, while the majority of cells in the injury site treated with Ad-*Dkk1* showed NICD2 staining. iAxin2/GFP IF confirmed successful Wnt inhibition. *n* = 3. Scale bar = 50 μm. **c**, **d** Notch target gene expression and **e** proliferative cell nuclear antigen (*Pcna)* gene expression from POD7 callus reveal activated Notch signaling in Ad-*Dkk1* treated injuries with increased proliferation (*n* = 3). **f** FACS analysis of SSCs in the fracture callus of Ad-*Null* and Ad-*Dkk1* treated animals at POD 3, 7, and 10 (*n* = 4) showing increase of SSC number during the proliferative phase after Wnt inhibition. **g** Primary bone marrow derived stromal cells were treated with PBS (control) or recombinant Dkk1 protein for 48 h in vitro. Hey1 IF shows an increased number of Notch activated cells after Dkk1 treatment. Experiments were performed in triplicate and repeated at least twice. **h** *Axin2* expression is decreased after Dkk1 treatment. **i** Resulting in increased *Hey1* gene expression (*n* = 3). PBS phosphate buffered saline. *p < 0.05, **p < 0.01, ***p < 0.001. Data were represented as mean ± s.e.m.

We hypothesize that Wnt signaling is upstream of Notch and regulates Notch signaling to initiate the switch from proliferation to differentiation. Using an adenovirus expressing the Wnt-antagonist *Dkk1*, we aimed to show that inhibition of Wnt signaling removes the inhibitory effect on Notch and thus maintains Notch activation. iAxin2/GFP mice were treated locally with either Ad-*null* or Ad-*Dkk1* at the time of tibial defect surgery (Fig. 3a). *Dkk1* treatment led to a significant reduction of *Axin2* expression in the regenerate, confirming successful Wnt inhibition (Fig. 3a). At postoperative day 7, we observed an increase in NICD2 (activated Notch2 intracellular domain) immunostaining in the injury sites treated with the Wnt antagonist *Dkk1* (Fig. 3b), indicating prolonged activated Notch signaling in the presence of a Wnt antagonist. In addition, *Hey1* and *Hes1* expression was increased in the Ad-*Dkk1* treated group at postoperative day 7 (Fig. 3c, d), suggesting that Wnt inhibition resulted in continued and increased Notch signal activation. If Notch is in fact regulating OPC proliferation, then we would expect to observe increased proliferation in the Ad-*Dkk1* treated injuries. We performed qRT-PCR for *proliferating cell nuclear antigen* (*Pcna*) and detected a significantly increased expression level in Ad-*Dkk1-*treated injury sites, suggesting that Wnt inhibition is sufficient to increase OPC proliferation through activation of Notch signaling, which functions as an activator of proliferation (Fig. 3e). If Wnt antagonism results in prolonged activated Notch signaling, then we should be able to detect an increase in the frequency of SSCs at the injury site after Wnt inhibition. We performed FACS analysis of the regenerate after Ad-Dkk1 treatment at POD 3, 7, and 10 and quantified the number of SSCs (Fig. 3f). There was no difference at day 3 between the control and Ad-*Dkk1* injuries, as one would expect as proliferation is just about to commence. At day 7 and 10, however, we observed a significant increase in SSC number in the Ad-Dkk1 treated injury sites, suggesting that Wnt inhibition results in Notch activation, which in turn results in increased and prolonged proliferation of SSCs in the injury site (Fig. 3f). We confirmed these in vivo findings using an in vitro assay of primary OPCs treated with Dkk1 protein or PBS. Immunofluorescence staining for Hey1 revealed increased nuclear Hey1 staining in Dkk1 treated OPCs (Fig. 3g). qRT-PCR confirmed that Dkk1 treatment resulted in Wnt inhibition, as shown by downregulation of *Axin2* (Fig. 3h). In response to Wnt inhibition, *Hey1* expression increased (Fig. 3i), confirming the hypothesis that Wnt signaling regulates Notch signaling in OPCs.

### Activated Wnt signaling inhibits Notch signaling and suppresses proliferation

**Fig. 4:**
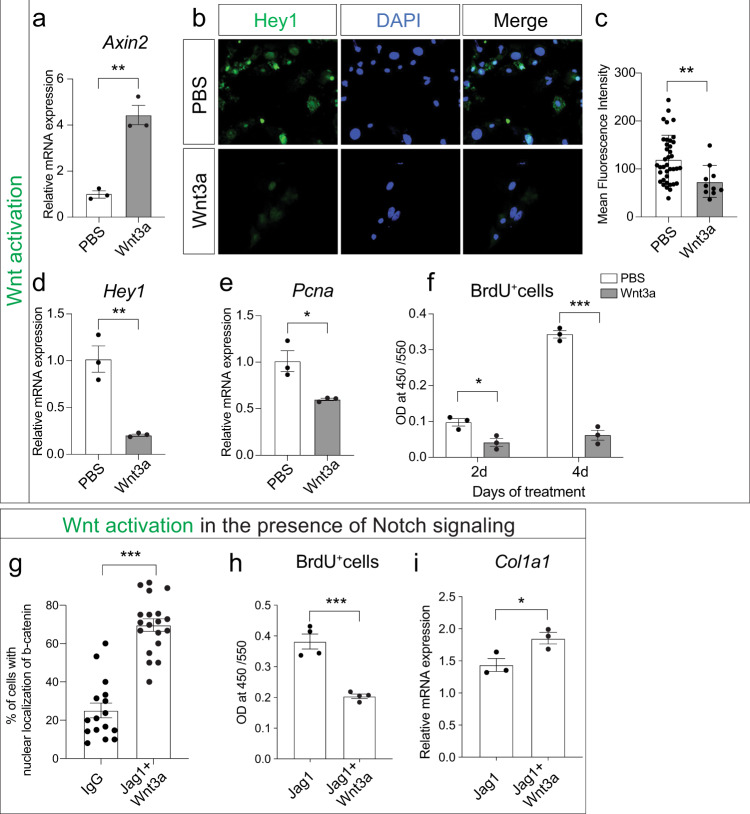
Wnt signal activation suppresses Notch and its associated proliferative effect. **a** *Axin2* gene expression is increased in progenitor cells treated with a recombinant protein Wnt3a (100 ng/ml) for 48 h in vitro. **b** Hey1 immunofluorescence, **c** quantification of Hey1 IF, and **d** gene expression of bone marrow stromal cells treated with PBS (control) or Wnt3a in vitro demonstrating Notch inhibition. **e**, **f** Decreased proliferation of bone marrow stromal cells after Wnt3a treatment shown by *Pcna* expression (**e**) and BrdU assay at 2 and 4 days in vitro (**f**). **g**–**i** OPCs were treated in vitro with Wnt3a in presence of Notch signaling (Jag1-coated plates) and analyzed for nuclear localization of β-catenin (**g**), BrdU-positive proliferating cells (**h**), and osteogenic differentiation marker *Col1a1* (**i**). *p < 0.05, **p < 0.01, ***p < 0.001. Experiments were performed in triplicate and repeated at least twice. Data were represented as mean ± s.e.m.

We further dissected the Notch–Wnt interaction using purified Wnt3a protein as a means to activate the Wnt pathway^44^. Bone marrow stromal cells were treated in vitro with Wnt3a protein, which resulted in Wnt pathway activation, as shown by *Axin2* expression (Fig. 4a). Immunohistochemistry for Hey1, a downstream target of the Notch signaling pathway, revealed decreased Hey1 expression after Wnt signal activation (Fig. 4b, c). This was confirmed by qRT-PCR (Fig. 4d), further establishing that Wnt pathway activation suppresses Notch signaling. If Notch is responsible for induction and maintenance of OPC proliferation, then we would expect decreased proliferation after Wnt activation. We quantified proliferative activity after Wnt3a treatment using two independent methods. qRT-PCR revealed decreased *Pcna* expression (Fig. 4e) and BrdU labeling demonstrated a 57% decrease in proliferation at day 2 after Wnt3a treatment and an 82% decrease at day 4 (Fig. 4f). Next, we aimed at investigating whether Wnt activation is able to downregulate activated Notch signaling. We treated bone marrow stromal cells grown on Jagged1-coated plates (Notch activation) with Wnt3a and observed an increase in the number of Wnt-responsive cells, shown by nuclear localization of beta-catenin (Fig. 4g). This increase in Wnt signal activity resulted in decreased proliferation (Fig. 4h) and increased differentiation (Fig. 4i). These data demonstrate that activated Wnt signaling can overcome activated Notch signaling, and that the upstream position of Wnt results in a switch from Notch-induced proliferation to Wnt-induced differentiation.

### In vivo inhibition of Wnt signaling prolongs Notch-induced proliferative phase

**Fig. 5:**
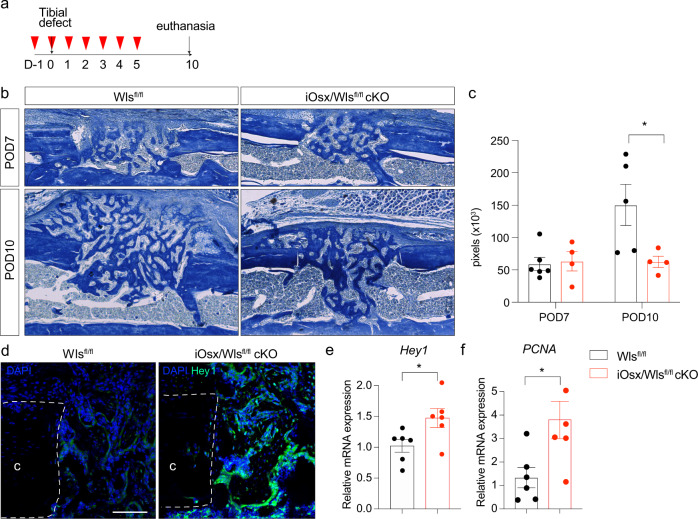
Wnt deletion in *osx*-lineage cells prolongs Notch responsiveness and associated proliferation. **a** Experimental scheme using iOsx/Wls^fl/fl^ cKO mice. Red arrow head indicates tamoxifen i.p. injection (1 mg/day), gray arrow denotes day of tibial defect surgery and day of euthanasia. **b** Aniline Blue staining of tibial defect sites at POD7 and POD10 in control and iOsx/Wls^fl/fl^ cKO mice. **c** Histomorphometry of bone volume in callus of control and iOsx/Wls^fl/fl^ cKO mice. **d** Increased and prolonged Notch responsiveness in iOsx/Wls^fl/fl^ cKO mice shown by IF against Hey1 and **e** *Hey1* gene expression. **f** PCNA expression revealed increased proliferation in the injury sites of iOsx/Wls^fl/fl^ cKO mice. Scale bar = 50 μm. *n* = 6. **d** *Pcna* expression at day 10 indicates a prolonged proliferative phase up to day 10 in the presence of decreased Wnt signaling. *p < 0.05. c cortical bone. Data were represented as mean ± s.e.m.

Finally, we utilized a genetic strategy to abrogate Wnt signaling during intramembranous bone healing using *Osx-cre*^*ERT2*^*/Wls*^*fl/fl*^ (iOsx/Wls^fl/fl^) conditional knockout mice^45^. Wntless (Wls) is a cargo receptor protein that directs Wnt ligands from the Golgi apparatus to the cell surface by interacting with the lipid-modified domains in the ligands^46–48^. We crossed *Osx-cre*^*ERT2*^ mice with *Wls*^*fl/f*l^ to generate an iOsx/Wls^fl/fl^ cKO that allows us to target Wnt secretion during defined stages of intramembranous bone healing. We injected Tamoxifen daily for 7 days in order to decrease Wnt ligand secretion within the injury site (Fig. 5a). First, we assessed the effect of decreased Wnt signaling on bone regeneration using histomorphometry. In line with the previously published and shown data, we observed impaired osteogenic differentiation with a significantly smaller bony regenerate at POD10 (Fig. 5b, c). Further analysis revealed that the conditional Wnt inactivation led to a prolonged Notch signal activation, shown by IF (Fig. 5d) qRT-PCR for *Hey1* confirmed the increased Notch downstream target expression after *Wls* deletion (Fig. 5e). In line with our previous data, the prolonged Notch activation resulting from Wnt inhibition led to a sustained proliferative response (Fig. 5f).

**Fig. 6:**
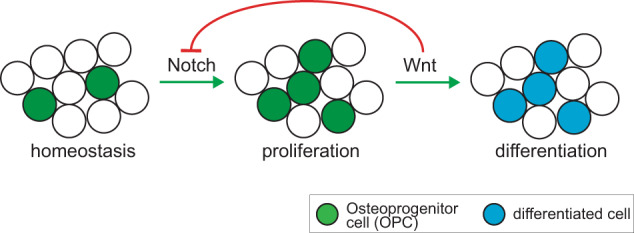
Graphical summary. After skeletal injury, Notch signaling becomes active and initiates a proliferative response. Days later, canonical Wnt signaling is activated in the injury site, leading to active suppression of Notch signaling and its associated pro-proliferative effect. Wnt signaling then induces differentiation.

In summary, our data provide convincing evidence that Notch signaling is active during early intramembranous bone healing, when Notch activation leads to osteoprogenitor cell proliferation. Once Wnt signaling becomes active, Notch signaling is inhibited, resulting in inhibition of proliferation and activation of differentiation (Fig. 6).

## Discussion

While many aspects of bone regeneration in response to fracture have been extensively characterized, certain facets are still unknown. One such knowledge gap is the molecular mechanism that orchestrates the switch from proliferation to differentiation. Here, using in vitro pathway manipulations and in vivo models of intramembranous bone healing, we provide evidence that proliferation in response to injury is regulated by Notch signaling and differentiation is initiated by Wnt signaling, which is, at the same time, inhibiting Notch signaling. In the most general terms, we uncovered the molecular machinery that regulates the transition from skeletal progenitor cell proliferation to osteogenic differentiation. We provide convincing evidence that Wnt signaling is necessary and sufficient to terminate progenitor cell proliferation and initiate osteogenic differentiation.

What is yet unknown is if there is a mechanism by which the cellular environment in the injury site senses the total number of OPCs available for differentiation. If such sensor exists, then the proliferative response period, required to establish a certain number of OPCs, would be unique for each injury site, rather than being defined by a molecular clock mechanism that switches off proliferation at an exact time after injury. Possible mechanisms by which OPC density could be detected is through cell–cell interaction, possibly between OPCs themselves or other surrounding cell types within the microenvironment of the early fracture callus. Another potential mechanism could be based on a growth factor gradient, similar to morphogens during development, which would instruct effector cells to switch from proliferation to differentiation. Wnt signaling is known to exert its effect through generation of a spatial gradient, and thus would be a prime candidate as a molecular switch regulating proliferation and differentiation. Future research will focus on identifying Wnt secreting cells within the injury site and testing whether their presence and spatial distribution is responsible for the increased Wnt activity around day 7 in the skeletal injury site, and if this signal activity is the molecular switch governing proliferative arrest and initiation of differentiation.

Antagonistic cross talk between the Notch and Wnt signaling pathways is not without precedent. Regulation of proliferation and differentiation during homeostasis and regeneration in the liver^49,50^ and intestine^51,52^ is controlled by the balance of these two pathways. In the zebrafish heart, Notch signaling is responsible for cardiomyocyte proliferation after injury, and artificial Wnt activation suppresses this Notch-related proliferative response to injury^34^. Our data now add to this in the biological context of skeletal regeneration where we propose that opposing Wnt and Notch signals regulate progenitor cell proliferation and subsequent differentiation into bone forming osteoblasts. Chen et al. previously demonstrated that β-catenin dependent Wnt signaling exerts disparate effects on mesenchymal cells and committed osteoblasts with an increase in TCF-dependent transcriptional activity during the initial phase of differentiation^39^. These data confirm our temporal expression analysis of Wnt responsiveness as well as corroborate the functional importance of Wnt signaling as the initiating activator of differentiation.

Our selection of the monocortical defect model to study the cellular and molecular events that regulate the transition from proliferation to differentiation was based on our experience that the temporal and spatial organization of the healing response is fairly organized and chronologic in this model, which could be considered a strength of this approach. However, results obtained from this study have to be evaluated under the assumption that this well-organized healing response of the monocortical defect model does not reflect the rather heterogenous environment of a fracture site, which usually heals through a mix of intramembranous and endochondral ossification. In particular, during endochondral ossification, Wnt and Notch signaling may play a different role than what was observed in this study, and most likely BMP signaling will play a more dominant role in the initiation of chondrogenic differentiation during the soft callus phase^53^. Our data presented herein, aim to elucidate the role of Notch and Wnt signaling during intramembranous ossification, and as such cannot be extrapolated to endochondral ossification. Yet, this approach may serve as a roadmap to study equal contributions of the signaling pathways during endochondral ossification.

In summary, we identified a molecular switch responsible for the transition of progenitor cell proliferation to differentiation during bone regeneration. These results will provide opportunities for potential translational approaches aimed at controlling proliferation and differentiation during fracture healing. If we could control the proliferative activity of OPCs after fracture, then we would be able to artificially expand the progenitor pool to a number necessary and sufficient to allow for successful bone healing. While Notch and Wnt signaling have been intensively studied individually, this paper is the first that links their function during tissue regeneration.

## Methods

### Animals

C57BL/6 mice (Jax no. 000664), B6.129(Cg)-*Axin2*^*tm1(cre/ERT2)Rnu*^/J (Axin2-creERT2, Jax no. 018867), *Gt(ROSA)26Sor*^*tm1.1(CAG-EGFP)Fsh*^/Mmjax (R26RGFP, Jax no. 32037-JAX), 129S-*Wls*^*tm1.1Lan*^/J (Wls^fl/fl^, Jax no. 012888), and B6.Cg-*Gt(ROSA)26Sor*^*tm14(CAG-tdTomato)Hze*^/J (tdTomato, Jax no. 007914) were purchased from Jackson Laboratory (Bar Harbor, ME). Floxed RBP-J mice (Rbpj^fl/fl^, RBRC No. RBRC01071)^36^ were purchased from Riken BioResource Center (Ibaraki, Japan). Osx-creERT2 mice were received from Dr. H. M. Kronenberg, Massachusetts General Hospital. To induce recombination in transgenic cre-ERT2 mice, tamoxifen (Sigma-Aldrich, St. Louis, MO, USA) was administered intraperitoneally either 1 mg/day for 7 consecutive days or single dose of 4 mg/day 1 day before euthanasia. Mice were maintained on a 12-h light/dark cycle with food and water provided ad libitum.

### Mono-cortical defects

All procedures followed protocols approved by the NYU Robert I. Grossman School of Medicine Committee on Animal Research. Mice were anaesthetized with an 1–4% Isoflurane inhalation. A 4-mm incision was made over the proximal anteromedial tibia, and the tibial surface was exposed while carefully preserving the periosteum. A 1.0-mm hole was drilled through the anterior cortex with a high-speed dental drill (10,000 rpm). Incisions were closed with 7–0 Vicryl sutures. Before and after surgery, mice received subcutaneous injections of buprenorphine for analgesia and were allowed to ambulate freely. Mice were euthanized at indicated days after surgery.

### Histology

After dissection, specimens were immediately fixed in 4% paraformaldehyde overnight at 4 °C and then decalcified in 19% ethylenediaminetetraacetic acid (EDTA) at 4 °C. Specimens were either paraffin embedded and cut into 10-μm-thick sections, or cryo embedded in a gelatin-based solution as described and cut into 50-μm-thick sections^54^. Pentachrome staining was performed on paraffin sections to identify osseous tissue as previously described^55^. Histochemistry for alkaline phosphatase (ALP) was conducted with BM Purple substrate (Roche, Indianapolis, IN, USA) according to the manufacturer’s instructions. Immunostaining was performed using Pcna (Cell Signaling Technology, #13110S, dilution 1:200, Danvers, MA, USA), Hey1 (Abcam, ab22614, dilution 1:100, Cambridge, MA, USA), Osterix (Abcam, ab22552, dilution 1:150), and cleaved N-terminus Notch2 (NICD2) (Millipore, 07-1234, Burlington, MA, USA). Sections were examined and photographed using a Leica digital imaging system (Leica, Wetzlar) or a Zeiss LSM 710 laser scanning confocal and multiphoton microscope (Carl Zeiss AG, Oberkochen, Germany).

Immunofluorescence staining was also performed in bone marrow cells cultured in chamber slides (Thermo Fisher Scientific, Waltham, MA, USA) using Hey1 (Abcam, dilution 1:200) and β-catenin (Abcam, ab32572, dilution 1:200).β-catenin positive nuclei were counted by imageJ software (US National Institutes of Health, Bethesda, MD, USA).

### μCT analyses

Samples were scanned using a high-resolution Skyscan μCT system (SkyScan 1172, Bruker, Billerica, MA). Images were acquired at 9 μm isotropic resolution using a 10MP digital detector,

About 10 W energy (100 kV and 100 A), and a 0.5 mm aluminum filter with a 9.7 μm image voxel size. A fixed global threshold method was used based on the manufacturer’s recommendations and preliminary studies, which showed that mineral variation between groups was not high enough to warrant adaptive thresholding. The following parameters were analyzed: total BV, total tissue volume (TV), respective mineralized volume fraction (BV/TV), following the guidelines described by Bouxsein et al.^56^. The volume of interest (VOI) included a region of the proximal tibia with the defect centered, including 1 mm proximal and distal to the injury site in order to capture periosteal callus formation outside of the 1 mm defect. The VOI was contoured to capture the entire callus region and total volume (TV) represents the entire callus volume within the above mentioned VOI.

### Isolation of skeletal progenitor cells from callus

Tibiae were harvested as previously described^57^. A 5 mm section including the injury site was excised and cells were isolated by mechanical and chemical digestion^27^. Briefly, each sample was crushed using mortar and pestle and subjected to enzymatic digestion with 0.2% collagenase at 37 °C under agitation. Cells were filtered through a 70-μm strainer, pelleted at 300 rcf at 4 °C. Red blood cells were lysed using NH_4_Cl (StemCell Technologies, Vancouver, Canada), washed with staining media (HBSS (Thermo Fisher Scientific) containing 2% FBS (Thermo Fisher Scientific), 1% HEPES (10 mM) (Thermo Fisher Scientific), and 1% penicillin-streptomycin (Thermo Fisher Scientific)) and pelleted.

### Flow cytometry

Dissociated cell samples were stained with antibodies against CD45, Ter119, Tie2, CD51, CD105, 6C3, CD90, and Notch receptors 1–4 and sorted by flow cytometry (Beckman-Coulter Moflo XDP, Brea, CA, USA; FACSAria^TM^ II, BD Biosciences, San Jose, CA, USA). CD45^−^Ter119^−^Tie2^−^CD51^+^CD90^−^6C3^−^CD105^+^ cells were identified as BCSPs^27–29^. All used antibodies for flow cytometry are listed in Table 1.

**Table 1:** Antibodies for flow cytometry.

Antibody	Company	Dilution
CD45-PE	Miltenyi Biotec	1:200
Ter119-PE	Miltenyi Biotec	1:200
Tie2-PE	Thermo Fisher Scientific	1:200
CD51-Biotin	Thermo Fisher Scientific	1:200
CD105-PE-Cy7	Thermo Fisher Scientific	1:200
6C3-PerCP-eFluor710	Thermo Fisher Scientific	1:200
6C3-PE	Thermo Fisher Scientific	1:200
CD90-FITC	Thermo Fisher Scientific	1:200
CD90-PE	Thermo Fisher Scientific	1:200
Streptavidin-APC	Thermo Fisher Scientific	1:200
Streptavidin-FITC	Thermo Fisher Scientific	1:200
Notch1-APC	Biolegend	1:200
Notch2-APC	Biolegend	1:200
Notch3-APC	Thermo Fisher Scientific	1:200
Notch4-APC	Biolegend	1:200
DAPI	Thermo Fisher Scientific	1:1000

### Isolation and culture of OPCs

For the in vitro experiments, tibial and femoral bone marrow cells were isolated by centrifugation from untreated 12-week-old C57BL/6 mice^58^. Cells were resuspended in growth media (DMEM (Thermo Fisher Scientific) containing 10% FBS and 1% penicillin-streptomycin) and then plated in 75-ml tissue culture flasks. The cells were trypsinized, seeded, and treated with Wnt3a 100 ng/ml (R&D, Minneapolis, MN, USA) or Dkk1 100 ng/ml (R&D) for 48 h. All cellular assays described below were performed at passage 1 from at least three different mice in three technical replicates.

### Proliferation assay

Cells were seeded in 96-well plates at a density of 2 × 10^4^ cells per well. BrdU assay (Abcam, Cambridge, UK) was performed according to the manufacturer’s instruction. Wells were read on a Flex Station 3 microplate reader (Molecular Devices, Sunnyvale, CA, USA) at 450 nm. Data were collected with Soft Max Pro (Molecular Devices) software. Means and standard error mean (SEM) were calculated in GraphPad Prism 8 software (GraphPad Software, Inc., La Jolla, CA, USA).

### RNA isolation and quantitative real-time PCR

RNA was isolated from either cells or callus using RNeasy Kit (Qiagen, Germantown, MD, USA) according to the manufacturer’s instruction. cDNA was synthesized using iScript^TM^ cDNA Synthesis Kit (Bio-Rad, Hercules, CA, USA). Quantitative real-time PCR was carried out using the Applied Biosystems Step One Plus detection system (Thermo Fisher Scientific) and RT2 SYBR Green ROX PCR Master Mix (Qiagen). Specific primers were designed using GETPrime 2.0^59^. Results are presented as 2^–ΔΔCt^ values normalized to the expression of 18 S. Means and SEMs were calculated in GraphPad Prism 8 software (Table 2).

**Table 2:** PCR primers.

Primer Name	Sequence (5′-3′)
18 S FOR	ACGAGACTCTGGCATGCTAACTAGT
18 S REV	CGCCACTTGTCCCTCTAAGAA
Hes1 FOR	TGCCAGCTGATATAATGGAG
Hes1 REV	CTTTGATGACTTTCTGTGCTC
Hes5 FOR	CGCATCAACAGCAGCATAGAG
Hes5 REV	TGGAAGTGGTAAAGCAGCTTC
Hey1 FOR	ACTACAGCTCCTCAGATAGTG
Hey1 REV	AACTCAAGTTTCCATTCTCGTC
Hey2 FOR	AGGGGGTAAAGGCTACTTTGA
Hey2 REV	TGGCGCAAGTGCTGAGATG
Axin2 FOR	CCTGGCTCCAGAAGATCAC
Axin2 REV	TAGGTGACAACCAGCTCAC
Pcna FOR	TGGAATCCCAGAACAGGAG
Pcna REV	TCAGAGCAAACGTTAGGTG
Col1a1 FOR	CAGTCGATTCACCTACAGCACG
Col1a1 REV	GGGATGGAGGGAGTTTACACG

All primers were purchased from Integrated DNA Technologies.

### RNAseq analysis

Libraries were sequenced on an Illumina HiSeq2500 sequencer. Sequencing results were demultiplexed and converted to FASTQ format using Illumina bcl2fastq software. The sequencing reads were aligned to the mouse genome (build mm10/GRCm38) using the splice-aware STAR aligner [http://www.ncbi.nlm.nih.gov/pubmed/23104886]. The featureCounts program [https://www.ncbi.nlm.nih.gov/pubmed/24227677] was utilized to generate counts for each gene based on how many aligned reads overlap its exons. These counts were then normalized and used to test for differential expression using negative binomial generalized linear models implemented by the DESeq2 R package [http://www.ncbi.nlm.nih.gov/pubmed/25516281].

### Adenovirus-mediated inhibition of Wnt signaling

Adenovirus expressing mouse *Dkk1* and CMV-*Null* (control) were purchased from Vector Biolabs (Malvern, PA, USA). Amplified adenovirus was purified using Adeno-X^TM^ Maxi Purification Kit and titered using Adeno-X^TM^ Rapid Titer Kit according to the manufacturer’s instruction (Takara Bio USA, Mountain View, CA, USA). Wnt inhibition was achieved locally by adsorbing 10^6^ ifu of adenovirus into 2 × 2 × 2-mm collagen sponge (DSI LTD, Azriqam settlement, Israel) and inserting the sponge under a muscle flap over the tibial defect site.

### Statistical analysis

A priori power analysis to obtain statistical significance (*p* = 0.05, power 80%) resulted in an *n* of 4 for each group, expecting a 25% difference between the groups. All cell culture-based assays were repeated at least three times and representative results were shown.

Prism 8 (GraphPad Software, Inc.) was used for statistical computations. A student’s *t*-test was used for all comparisons in which there were two groups; ANOVA analyses followed by the Holms–Sidak correction for post hoc testing was applied for analyses in which there were two or more comparisons being made. Error bars represent SEMs. *P* < 0.05 was considered to be statistically significant. An asterisk symbol (*) denotes a *p* value < 0.05, unless denoted otherwise in figure legend.

### Reporting Summary

Further information on research design is available in the Nature Research Reporting Summary linked to this article.

## Supplementary information

Supplementary Information

Reporting Summary

## Data Availability

The data that support the findings of this study are available from the corresponding author upon reasonable request. The RNA sequencing data are deposited in GEO (GSE173371).
