# Supplementary material for: Notch-Wnt signal crosstalk regulates proliferation and differentiation of osteoprogenitor cells during intramembranous bone healing
Source: NPJ Regen Med. 2021 May 28;6:29. doi: 10.1038/s41536-021-00139-x (PMC8163848; doi:10.1038/s41536-021-00139-x)
Supplement: Supplementary file 1 — Supplementary Information [file 41536_2021_139_MOESM1_ESM.pdf]

Supplemental Figure 1

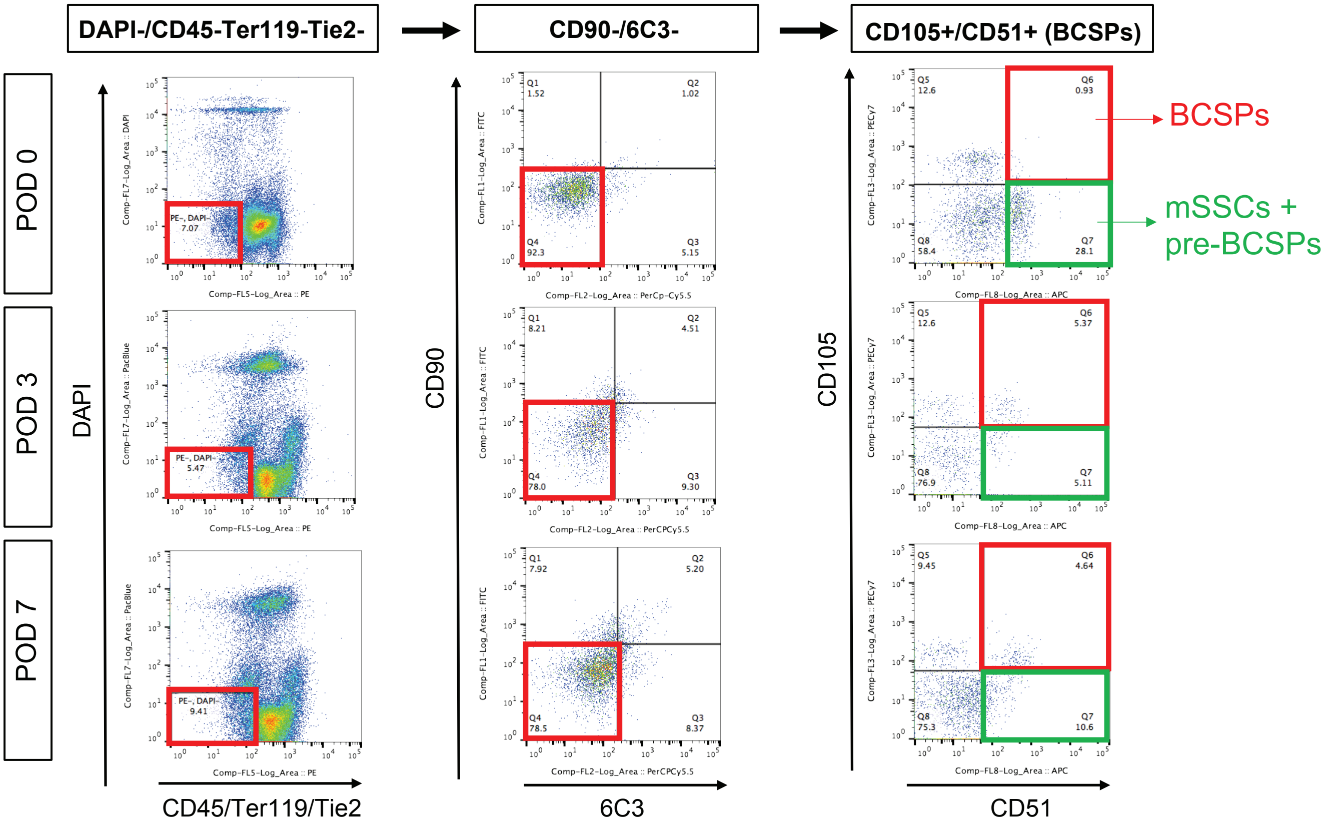

Supplementary Figure 1. Gating strategy for SSC and BCSP analyses. Frequency of SSCs and BCSPs was analyzed at the indicated post-operative day using this gating strategy in figures 1-3.

Supplemental Figure 2

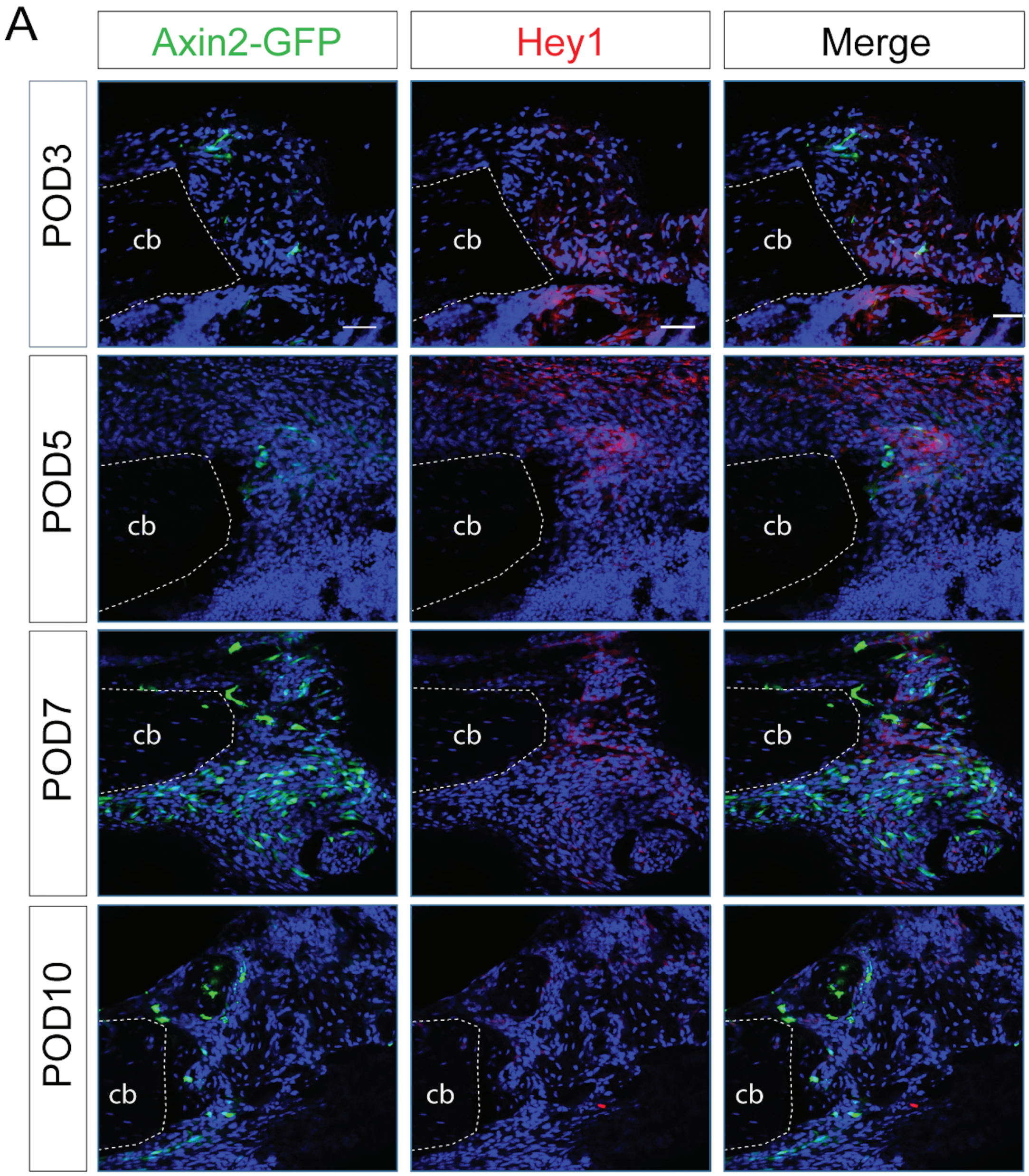

Supplementary Figure 2. Temporospatial Notch and Wnt downstream target activity. **(A)** Immunofluorescence of tibial defect injury sites at POD 3-10 demonstrating Notch downstream target Hey1 expression during the early proliferative phase of repair, while the Wnt downstream target Axin2 is expressed during the later differentiation phase (n=4).

Supplemental Figure 3

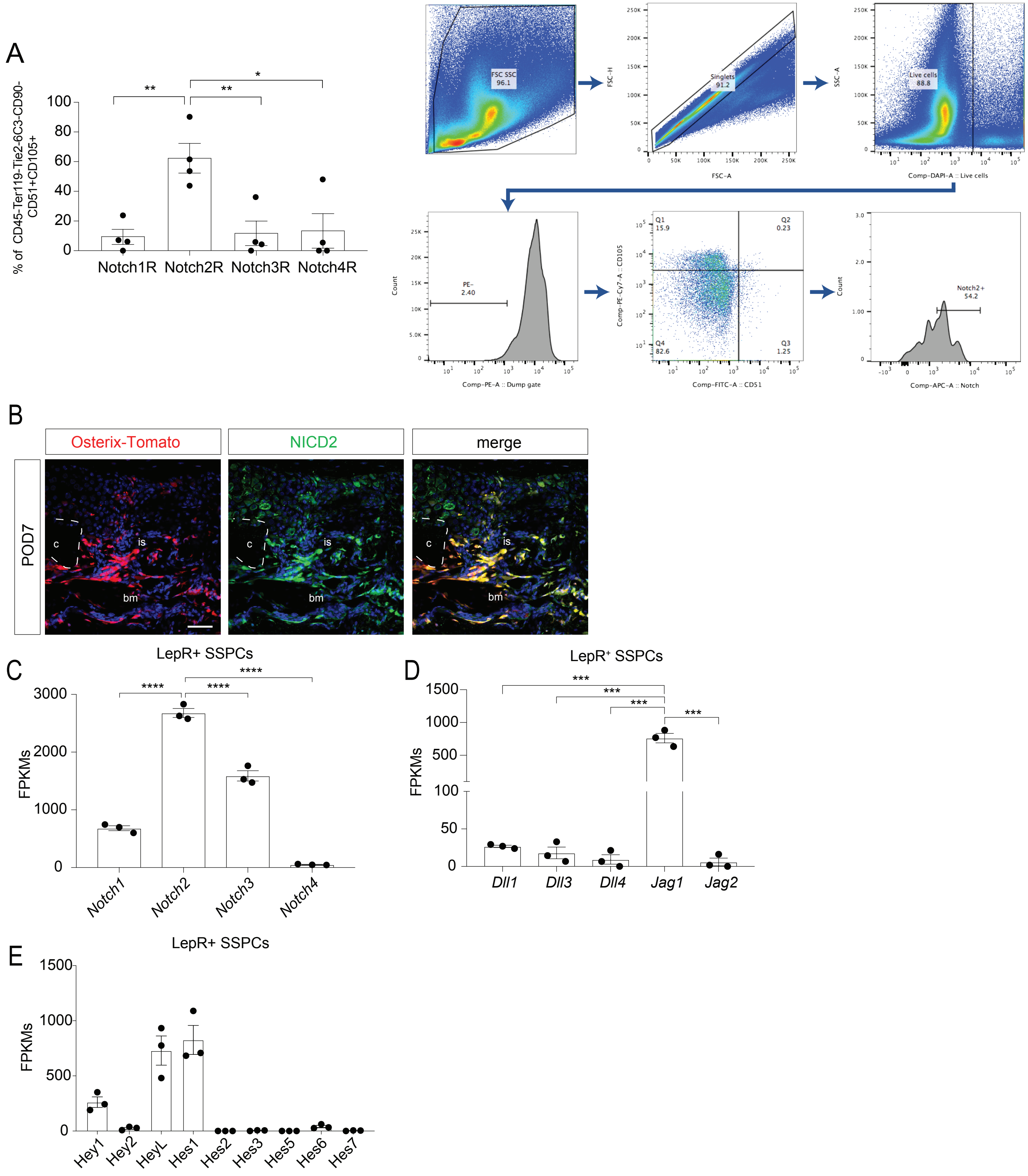

Supplementary Figure 3. Notch 2 receptors are the most abundant in osteoprogenitor cells among Notch receptors. **(A)** Frequency of Notch 1-4 receptors in BCSPs were analyzed by flow cytometry (representative gating strategy). **(B)** Immunofluorescence staining of activated Notch 2 receptor and Osterix-lineage cells at day 7 after tibial defects. **(C)** RNAseq analysis of LepR+ skeletal stem and progenitor cells showing expression of Notch 1-4 **(D)** Notch ligand expression, and **(E)** Notch downstream targets. Scale bar = 50μm. Abbr.: c, cortical bone; is, injury site; bm, bone marrow; POD, post-operative day. Data are represented as mean ± s.e.m.
