# Supplementary material for: Notch-Wnt signal crosstalk regulates proliferation and differentiation of osteoprogenitor cells during intramembranous bone healing
Source: NPJ Regen Med. 2021 May 28;6:29. doi: 10.1038/s41536-021-00139-x (PMC8163848; doi:10.1038/s41536-021-00139-x)
Supplement: Supplementary file 2 — Reporting Summary [file 41536_2021_139_MOESM2_ESM.pdf]

nature research

Corresponding author(s): Philipp Leucht

Last updated by author(s): 4/26/2021

# Reporting Summary

Nature Research wishes to improve the reproducibility of the work that we publish. This form provides structure for consistency and transparency in reporting. For further information on Nature Research policies, see our Editorial Policies and the Editorial Policy Checklist.

## Statistics

For all statistical analyses, confirm that the following items are present in the figure legend, table legend, main text, or Methods section.

| n/a | Confirmed | |
|---|---|---|
| ☐ | ☒ | The exact sample size (*n*) for each experimental group/condition, given as a discrete number and unit of measurement |
| ☐ | ☒ | A statement on whether measurements were taken from distinct samples or whether the same sample was measured repeatedly |
| ☐ | ☒ | The statistical test(s) used AND whether they are one- or two-sided<br>*Only common tests should be described solely by name; describe more complex techniques in the Methods section.* |
| ☐ | ☒ | A description of all covariates tested |
| ☐ | ☒ | A description of any assumptions or corrections, such as tests of normality and adjustment for multiple comparisons |
| ☐ | ☒ | A full description of the statistical parameters including central tendency (e.g. means) or other basic estimates (e.g. regression coefficient) AND variation (e.g. standard deviation) or associated estimates of uncertainty (e.g. confidence intervals) |
| ☐ | ☒ | For null hypothesis testing, the test statistic (e.g. *F*, *t*, *r*) with confidence intervals, effect sizes, degrees of freedom and *P* value noted<br>*Give P values as exact values whenever suitable.* |
| ☒ | ☐ | For Bayesian analysis, information on the choice of priors and Markov chain Monte Carlo settings |
| ☒ | ☐ | For hierarchical and complex designs, identification of the appropriate level for tests and full reporting of outcomes |
| ☐ | ☒ | Estimates of effect sizes (e.g. Cohen's *d*, Pearson's *r*), indicating how they were calculated |

*Our web collection on statistics for biologists contains articles on many of the points above.*

## Software and code

Policy information about availability of computer code

| | |
|---|---|
| Data collection | Not relevant for this publication |
| Data analysis | Not relevant for this publication |

For manuscripts utilizing custom algorithms or software that are central to the research but not yet described in published literature, software must be made available to editors and reviewers. We strongly encourage code deposition in a community repository (e.g. GitHub). See the Nature Research guidelines for submitting code & software for further information.

## Data

Policy information about availability of data

All manuscripts must include a data availability statement. This statement should provide the following information, where applicable:

- Accession codes, unique identifiers, or web links for publicly available datasets
- A list of figures that have associated raw data
- A description of any restrictions on data availability

The data that support the findings of this study are available from the corresponding author upon reasonable request.

# Field-specific reporting

Please select the one below that is the best fit for your research. If you are not sure, read the appropriate sections before making your selection.

☒ Life sciences ☐ Behavioural & social sciences ☐ Ecological, evolutionary & environmental sciences

For a reference copy of the document with all sections, see nature.com/documents/nr-reporting-summary-flat.pdf

# Life sciences study design

All studies must disclose on these points even when the disclosure is negative.

| | |
|---|---|
| Sample size | A priori power analysis to obtain statistical significance (p=0.05, power 80%) resulted in an n of 4 for each group, expecting a 25% difference between the groups. All cell culture–based assays were repeated at least three times and representative results were shown. |
| Data exclusions | No data were excluded |
| Replication | Experimental replication was performed as stated in the manuscript text. |
| Randomization | Animals were randomized to treatment groups. |
| Blinding | All analysis were performed or confirmed by investigators blinded to the experimental conditions. |

# Reporting for specific materials, systems and methods

We require information from authors about some types of materials, experimental systems and methods used in many studies. Here, indicate whether each material, system or method listed is relevant to your study. If you are not sure if a list item applies to your research, read the appropriate section before selecting a response.

## Materials & experimental systems

| n/a | Involved in the study | |
|---|---|---|
| ☐ | ☒ | Antibodies |
| ☒ | ☐ | Eukaryotic cell lines |
| ☒ | ☐ | Palaeontology and archaeology |
| ☐ | ☒ | Animals and other organisms |
| ☒ | ☐ | Human research participants |
| ☒ | ☐ | Clinical data |
| ☒ | ☐ | Dual use research of concern |

## Methods

| n/a | Involved in the study | |
|---|---|---|
| ☒ | ☐ | ChIP-seq |
| ☐ | ☒ | Flow cytometry |
| ☒ | ☐ | MRI-based neuroimaging |

## Antibodies

| | |
|---|---|
| Antibodies used | Pcna (Cell Signaling Technology, #13110S, dilution 1:200, Danvers, MA, USA), Hey1 (Abcam, ab22614, dilution 1:100, Cambridge, MA, USA), Osterix (Abcam, ab22552, dilution 1:150), and cleaved N-terminus Notch2 (NICD2) (Millipore, 07-1234, Burlington, MA, USA). |
| Validation | Commercial antibodies which are validated by referenced publications on the manufacturer's website and in several independent publications. |

## Animals and other organisms

Policy information about studies involving animals; ARRIVE guidelines recommended for reporting animal research

| | |
|---|---|
| Laboratory animals | C57BL/6 mice (Jax no. 000664), B6.129(Cg)-Axin2tm1(cre/ERT2)Rnu/J (Axin2-creERT2, Jax no. 018867), Gt(ROSA)26Sortm1.1(CAG-EGFP)Fsh/Mmjax (R26RGFP, Jax no. 32037-JAX), 129S-Wlstm1.1Lan/J (Wlsfl/fl, Jax no. 012888), and B6.Cg-Gt(ROSA)26Sortm14(CAG-tdTomato)Hze/J (tdTomato, Jax no. 007914) were purchased from Jackson Laboratory (Bar Harbor, ME). Floxed RBP-J mice (Rbpjfl/fl, RBRC No. RBRC01071)36 were purchased from Riken BioResource Center (Ibaraki, Japan). Osx-creERT2 mice were received from Dr. H. M. Kronenberg, Massachusetts General Hospital. |
| Wild animals | n/a |
| Field-collected samples | n/a |
| Ethics oversight | n/a |

Note that full information on the approval of the study protocol must also be provided in the manuscript.

# Flow Cytometry

## Plots

Confirm that:

☒ The axis labels state the marker and fluorochrome used (e.g. CD4-FITC).

☒ The axis scales are clearly visible. Include numbers along axes only for bottom left plot of group (a 'group' is an analysis of identical markers).

☒ All plots are contour plots with outliers or pseudocolor plots.

☒ A numerical value for number of cells or percentage (with statistics) is provided.

## Methodology

| | |
|---|---|
| Sample preparation | Dissociated cell samples were stained with antibodies against CD45, Ter119, Tie2, CD51, CD105, 6C3, CD90 and Notch receptors 1-4. |
| Instrument | Beckman-Coulter Moflo XDP, Brea, CA, USA; FACSAriaTM II, BD Biosciences, San Jose, CA, USA |
| Software | FlowJo |
| Cell population abundance | see suppl. fig |
| Gating strategy | see suppl. fig |

☒ Tick this box to confirm that a figure exemplifying the gating strategy is provided in the Supplementary Information.
